# Preface: the ecology and evolution of bacterial immune systems

**DOI:** 10.1098/rstb.2024.0064

**Published:** 2025-09-04

**Authors:** Edze Rients Westra, Uri Gophna, Stineke van Houte

**Affiliations:** ^1^ Department of Biosciences, University of Exeter, Exeter EX4 4QD, UK; ^2^ Molecular Microbiology and Biotechnology Department, Tel Aviv University, Tel Aviv-Yafo, Israel

**Keywords:** evolution, ecology, bacteria, phage

The constant exposure of bacteria to viral parasites, known as bacteriophages (phages), has driven the evolution of a huge number of bacterial immune mechanisms that can protect against these infections. Over the past decade, the landscape of bacterial immunity has been radically transformed by a surge of discoveries. These have revealed an astonishing diversity of defensive mechanisms, many of which operate via entirely novel molecular principles. Interestingly, as discussed in a perspective by Barthes *et al*. [1] in this Discussion Meeting issue, many of these immune strategies are ancient and in some cases conserved across domains of life. Their discovery and characterization therefore help us to identify general principles of host defence against viral infections.

Though our understanding of the mechanisms, ecology and evolution of immune systems is progressing, we are still facing numerous unanswered questions. This issue brings together researchers from across disciplines to address some of these questions. Contributions range from comparative genomics and structural biology to experimental evolution and environmental ecology, all aimed at illuminating the principles that govern immune system diversity, function and evolution. By uniting these diverse perspectives, we hope to foster a more integrated understanding of how bacteria defend themselves against viral predators, and how such defence mechanisms are shaped by the environments in which they evolve.

The issue covers the following areas. First, a number of contributions address gaps in our mechanistic understanding of immune systems. For many defence strategies, we still lack answers to some of the most fundamental questions: How are immune responses regulated? What are the molecular sensors that detect phage infection and initiate the immune response? What specific molecular features, such as modified bases in viral DNA or host functionalities that are essential for phage replication, do these systems recognize and target? And once a response has successfully been launched, how does the system ‘reset’ after the infection has been cleared? Here, Koonce *et al*. [2] describe the identification of regulators of the CRISPR-Cas immune system of *Pseudomonas aeruginosa*, while Readshaw *et al*. [3] and Belukhina *et al*. [4] identify the targets of type IV restriction–modification (RM) and PARIS immune systems, respectively, and Hoikkala *et al.* [5] describe the identification and distribution of enzymes involved in the deactivation of the type III CRISPR-Cas immune system.

Second, we have an incomplete understanding of the phenotypes that immune systems produce. Some systems, like RM, cleave the phage genome, whereas other systems, such as CBASS, are thought to trigger cell death or dormancy. These phenotypes may vary depending on the genetic background of the host bacterium, the nature of the infecting phage and the presence or absence of other immune systems. In this theme issue, Clabby *et al*. [6] present a perspective on the mechanisms that may drive defence co-occurrence within bacterial genomes, and the potential redundancy, synergy or division of labour between different systems. Kupczok *et al.* [7] describe computational methods to link bacterial genotypes to phage resistance phenotypes, and Pons *et al.* [8] present single-cell analysis of bacteria with either CRISPR immunity or receptor mutations, to identify genotype–phenotype links at a resolution not achievable in bulk assays. David *et al.* [9] experimentally examine how different defence systems of *Escherichia coli* isolates contribute to their resistance profile against phage infections, using systematic knock-outs of different defence systems, whereas Czernuszka *et al.* [10] examine how the number of defence systems per genome predicts the infectivity/resistance profile of phage cocktails against a large panel of clinical *P. aeruginosa* isolates.

Third, we know surprisingly little about how different defences are distributed across ecosystems, and how environments select for specific immune systems. In this issue, Meaden *et al.* [11] use metagenomics data analysis to determine how defence systems are distributed across different ecological niches, and how this covaries with the risk of phage infection. Silk *et al.* [12] use experimental evolution approaches to examine the impact of abiotic variables, such as salt concentrations, on the molecular evolution of phage resistance. Elliot *et al.* [13] examine how the protective ability of CRISPR-Cas immune systems varies depending on the phage and nutrient levels in the environment.

At a larger scale, defence systems may drive the evolution and epidemiology of bacterial pathogens. Blokesch [14] reviews the conserved defence mechanisms of the seventh pandemic El Tor (7PET) strain of *Vibrio cholerae* and their association with mobile genetic elements, and Blokesch & Seed [15] explain how coevolution between *V. cholerae* and its phage shapes cholera epidemiology. Le Roux [16] reviews how other *Vibrio* species can serve as powerful models to study the eco-evolutionary dynamics of bacteria and phage interactions and how bacterial defence systems are frequently moved around by mobile genetic elements themselves. Finally, Ojiogu *et al*. [17] describe strategies that mobile genetic elements can use to combat one another, identifying a novel mechanism used by *Staphylococcus aureus* pathogenicity islands to redirect helper phage capsid assembly. This highlights the key point of conflict between mobile genetic elements and how various mechanisms may have evolved not only to protect the bacterial hosts but also to outcompete other mobile genetic elements that can infect the same strain or species, a point that is further discussed in the perspective by Clabby *et al*. [6].

As the bacterial immune system landscape continues to expand, the field stands at an exciting and challenging juncture. We now face the dual tasks of filling in the mechanistic gaps in our understanding, while also zooming out to appreciate how these systems function and evolve within microbial ecosystems. This iscussion Meeting issue aims to contribute meaningfully to both of these goals.

## Data Availability

This article has no additional data.
